# Tissue distribution and urinary excretion of intravenously administered chemically functionalized graphene oxide sheets†
†Electronic supplementary information (ESI) available. See DOI: 10.1039/c5sc00114e


**DOI:** 10.1039/c5sc00114e

**Published:** 2015-04-14

**Authors:** Dhifaf A. Jasim, Cécilia Ménard-Moyon, Dominique Bégin, Alberto Bianco, Kostas Kostarelos

**Affiliations:** a Nanomedicine Laboratory , Faculty of Medical & Human Sciences and National Graphene Institute , University of Manchester , AV Hill Building , Manchester M13 9PT , UK . Email: kostas.kostarelos@manchester.ac.uk; b CNRS , Institut de Biologie Moléculaire et Cellulaire , Laboratoire d'Immunopathologie et Chimie Thérapeutique , 67000 Strasbourg , France . Email: a.bianco@ibmc-cnrs.unistra.fr; c Institut de Chimie et Procédés pour l'Energie , l'Environnement et la Santé (ICPEES) , ECPM , UMR 7515 du CNRS , University of Strasbourg , 25 rue Becquerel Cedex 02 , 67087 Strasbourg , France

## Abstract

The design of graphene-based materials for biomedical purposes is of great interest. Graphene oxide (GO) sheets represent the most widespread type of graphene materials in biological investigations. In this work, thin GO sheets were synthesized and further chemically functionalized with DOTA (1,4,7,10-tetraazacyclododecane-1,4,7,10-tetraacetic acid), a stable radiometal chelating agent, by an epoxide opening reaction. We report the tissue distribution of the functionalized GO sheets labeled with radioactive indium (^111^In) after intravenous administration in mice. Whole body single photon emission computed tomography (SPECT/CT) imaging, gamma counting studies, Raman microscopy and histological investigations indicated extensive urinary excretion and predominantly spleen accumulation. Intact GO sheets were detected in the urine of injected mice by Raman spectroscopy, high resolution transmission electron microscopy (HR-TEM) and electron diffraction. These results offer a previously unavailable pharmacological understanding on how chemically functionalized GO sheets transport in the blood stream and interact with physiological barriers that will determine their body excretion and tissue accumulation.

## Abstract 2

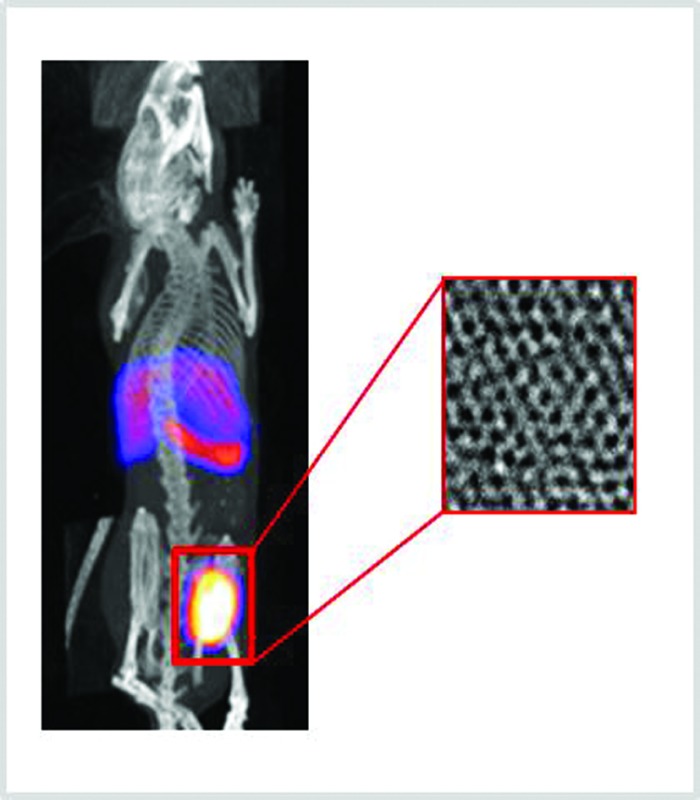
Providing a pharmacological understanding on how chemically functionalized GO sheets transport in the blood stream and interact with physiological barriers that determine their body excretion and tissue accumulation.

## Introduction

Graphene and related materials exhibit outstanding properties generated from their unique 2D carbon geometry.^1^,^2^ These properties have attracted great interest from different scientific disciplines, ranging from physics, materials science and more recently biomedicine.^2^–^5^ One of the most interesting properties for biomedical applications is their large available surface area that provides an ideal platform for bio-functionalization with small therapeutic molecules, macromolecules and imaging probes by covalent attachment or physisorption.^6^–^8^ The high mechanical strength^9^–^11^ and flexibility of graphene materials^2^,^3^ offer interesting and largely unexplored possibilities on interaction with soft biological matter,^10^,^12^ some of which are directly relevant to regenerative medicine and prosthetic applications.^2^

Graphene oxide (GO) is only one type in the graphene family of nanomaterials.^13^,^14^ Even though it suffers from compromised electronic properties, due to the extensive surface defects caused by oxidation,^7^,^15^ it has been extensively explored in the biological context. The availability of several types of oxygenated groups on the edge and planar surfaces of GO facilitates dispersibility and stability in physiological environments that affords improved biocompatibility.^6^

In view of the potential of using graphene materials for biomedical applications, it is critical to understand its interaction and fate *in vivo*.^16^ GO prepared by the modified Hummers' method has been administered intravenously,^17^–^19^ intraperitoneally,^20^,^21^ orally^21^ and intravitreally^22^ with no reported cytotoxic responses, histopathological changes or inflammatory reaction, even after long exposure time points.^21^,^22^ After intravenous or intraperitoneal administration, GO has been reported to accumulate mainly in the reticuloendothelial system (RES) organs with slow clearance over time.^17^,^23^ Direct lung administration by intratracheal, intrapleural or pharyngeal aspiration of pristine graphene, GO or reduced GO (rGO) resulted in lung retention with activation of the acute and chronic inflammatory pathways and lung injury.^24^–^26^ However, no biopersistance was seen with slow clearance in the mediastinal lymph nodes over time.^25^ Moreover, *in vivo* degradation of intravenously injected functionalized graphene three months post-injection has been reported in tissue residing macrophages, mainly in the spleen.^27^

It must be emphasized that all the above mentioned studies have used different types of GO,^14^,^28^ which can result in sharply different biological interactions.^29^,^30^ These interactions will be dependent on the type of surface functionalization, functional groups^31^,^32^ and dimensions of the GO sheets.^17^ Here, we report whole body imaging and pharmacokinetic data following intravenous administration of DOTA-functionalized GO (GO–DOTA) coupled with analytical and histopathological analysis of critical organs using individualized stable dispersions of thin GO sheets.

## Results

### Preparation and characterization of GO and GO–DOTA

**Scheme 1:**
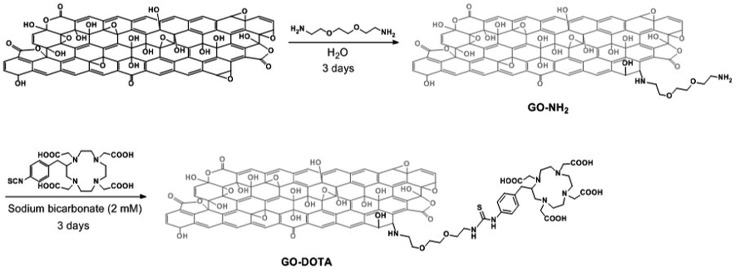
Preparation of GO–DOTA by a two-step derivatization method. For the sake of clarity, only one epoxide group is derivatized.

GO was prepared by the modified Hummers' method as described previously.^20^ To allow the grafting of DOTA, GO was initially derivatized with amine functions (Scheme 1). For this purpose, we exploited the presence of epoxy functions on the basal plane of GO,^33^ which are highly reactive towards nucleophiles.^34^,^35^ We used triethylene glycol (TEG) diamine to open the epoxy rings and introduce amino functions on GO. The TEG chain preserved the aqueous dispersibility of GO. The amount of amino groups on GO–NH_2_ was assessed by the Kaiser test.^36^ The loading corresponded to 670 μmol of NH_2_ functions per gram of GO. Then, the amino groups of GO–NH_2_ were derivatized with a DOTA derivative bearing an isothiocyanate moiety (DOTA–NCS). The Kaiser test of GO–DOTA indicated that the amount of unreacted amine functions was 315 μmol g^–1^, accounting for an ∼50% coupling efficiency.

**Fig. 1:**
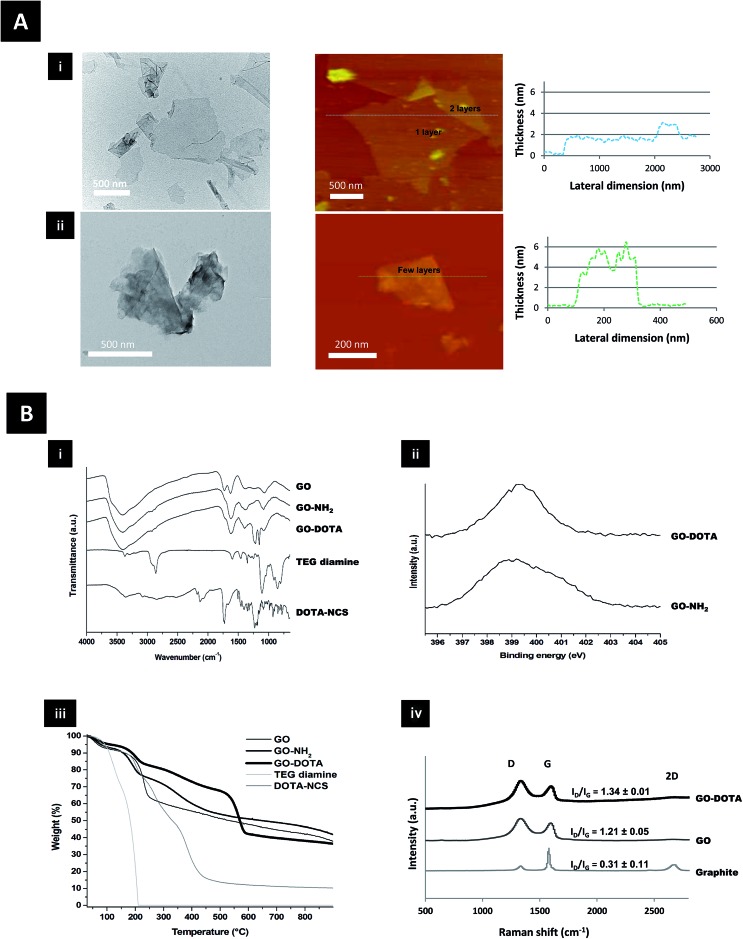
(A) Structural characterization of GO (i) and GO–DOTA (ii) by TEM (left), AFM and AFM-height section (right). (B) Physicochemical characterization using (i) FT-IR spectroscopy of GO, GO–NH_2_, GO–DOTA, TEG diamine, and DOTA–NCS; (ii) XPS N (1 s) peak of GO–NH_2_ and GO–DOTA; (iii) TGA of GO, GO–NH_2_, GO–DOTA, TEG diamine, and DOTA–NCS under inert atmosphere; (iv) Raman spectroscopy of graphite, GO, and GO–DOTA, with corresponding *I*_D_/*I*_G_ ratio.

Structural characterization of both GO–NH_2_ and GO–DOTA is shown in Fig. 1 using transmission electron microscopy (TEM) and atomic force microscopy (AFM). The AFM height sections (Fig. 1A and S1†) revealed that the thickness of the GO sheets was increased from single to a few layers after functionalization, while the size distribution was moderately reduced. DOTA conjugation onto GO was further studied by Fourier transform infrared (FT-IR) spectroscopy, X-ray photoelectron spectroscopy (XPS), thermogravimetric analysis (TGA) and Raman spectroscopy (Fig. 1B and S2†). The appearance of new bands in the FT-IR spectra of GO–NH_2_ and GO–DOTA evidenced the chemical modification of GO. XPS confirmed the presence of nitrogen after derivatization with TEG diamine and DOTA–NCS. TGA under inert atmosphere allowed us to determine the thermal profile of GO and functionalized GO, as the functional groups on the graphene surface are thermally labile. The different changes observed were indicative of covalent functionalization of GO. The *I*_D_/*I*_G_ ratios of GO derivatives obtained from the Raman spectra were increased in comparison to the starting graphite, as the samples were covalently functionalized.

### Radiolabeling efficiency and stability of GO–DOTA[^111^In]

The efficiency of radiolabeling GO–DOTA was compared to a physically adsorbed control (GO + DOTA), prepared by simple mixing of GO with DOTA (Scheme S1†), and to controls (DOTA[^111^In] and EDTA[^111^In]). As shown in Fig. S3A,† the radiolabeling efficiency of the covalent sample was found to be double that of the physically adsorbed control. Removal of unbound DOTA[^111^In] and EDTA[^111^In] was performed by centrifugation and no free ^111^In label or DOTA was detected in the covalent sample as displayed in Fig. S3B.† This was also confirmed before injecting the samples into C57BL/6 mice (Fig. S3C†). DOTA conjugation efficiency was further analysed using a modified running buffer at high pH (pH 9). At this pH, free ^111^In precipitates at the application point, while any chelator (DOTA) bound ^111^In runs to the solvent front. Fig. S3D† revealed no free DOTA chelator in the sample, confirming further the DOTA conjugation.

The radiolabeling stability of GO–DOTA[^111^In] and GO + DOTA[^111^In] in both 50% serum and PBS at 37 °C up to 24 h is shown in Fig. S3E.† The physically adsorbed sample (GO + DOTA) showed release of DOTA over time, with 50% release in serum, while the covalent sample was stable up to 24 h. Similar results were obtained when simply mixing GO with ^111^In in the absence of DOTA, where the labeling was both unstable and non-efficient (data not shown). Furthermore, the colloidal stability of the GO–DOTA was studied in different dispersion media, including dextrose 5%, PBS and 50% serum (Fig. S4†). GO–DOTA sheet dispersions were very stable in dextrose and serum-containing media, however aggregation and an increase in mean particle diameter was detected in the PBS environment very rapidly, suggesting electrostatic destabilisation.

### Pharmacokinetics and tissue distribution after intravenous administration

**Fig. 2:**
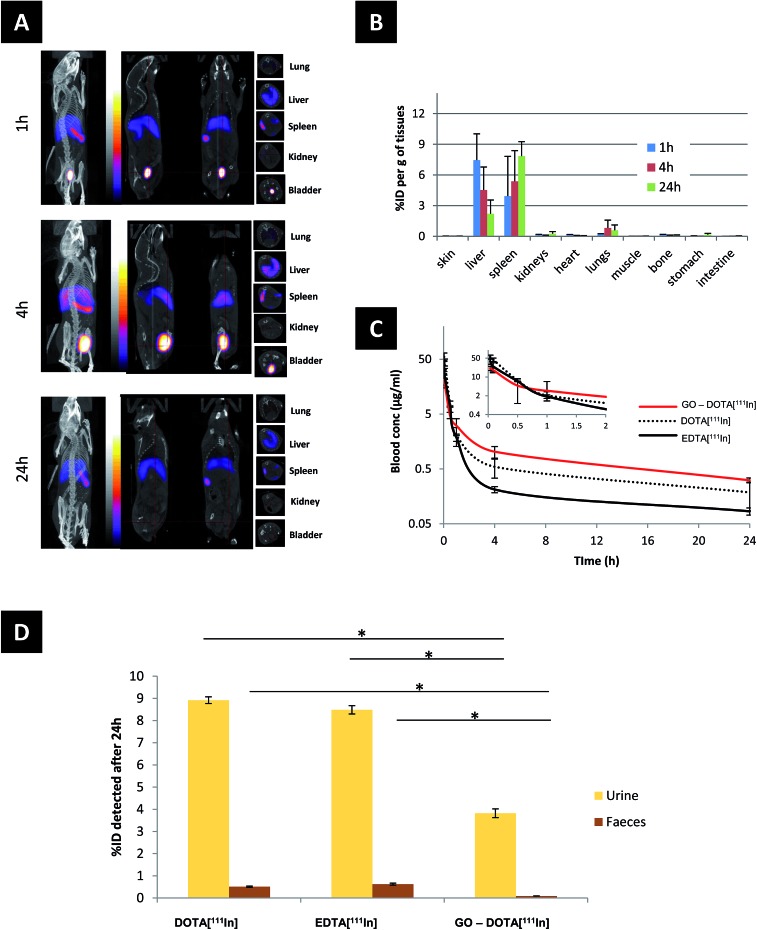
(A) Whole body Nano-SPECT/CT imaging of a C57BL/6 mouse injected with 50 μg of GO–DOTA[^111^In], imaged at different time points (1, 4, and 24 h), showing from left to right whole body, sagittal, coronal and transverse views. (B) Major organ biodistribution, (C) blood profile, and (D) levels of radioactivity detected in urine and faeces after 24 h detected by gamma scintigraphy. Statistical significance was * <0.05 against both controls using Student's *t*-test. Four different mice were used per group.

The biodistribution of GO–DOTA[^111^In] by SPECT/CT imaging and cut-and-count γ-scintigraphy is displayed in Fig. 2A–D. Rapid clearance from blood was detected within the first hour following administration (Fig. 2C) with a strong bladder signal, along with spleen and liver uptake (Fig. 2A). The whole-body SPECT/CT imaging data were validated by counting the percentage of the injected dose of GO–DOTA[^111^In] per gram of tissue measured by gamma scintigraphy in a separate experiment (Fig. 2B). At 4 h post-injection, a stronger signal was detected in the bladder by SPECT/CT, indicating further urinary excretion. This was in agreement with the significant quantity that was detected in the pooled urine samples after 24 h (Fig. 2D) using metabolic cages. Both controls DOTA[^111^In] and EDTA[^111^In] showed almost complete clearance within the first hour, by both SPECT/CT imaging and γ-scintigraphy (Fig. S5†).

**Fig. 3:**
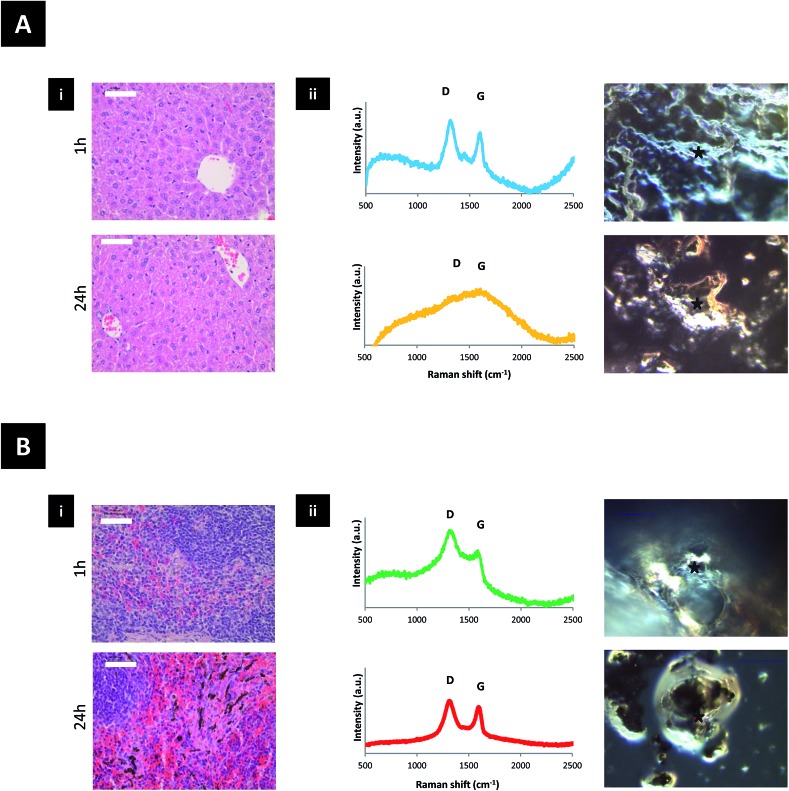
Translocation events in mice injected with 50 μg of GO–DOTA chelated with non-radioactive InCl_3_ after 1 and 24 h. (A) Liver and (B) spleen examinations using (i) H & E staining and (ii) Raman spectroscopy (average of 3 spectra) (left) and corresponding optical micrographs (right). All scale bars are 50 μm.

Translocation of the signal from the liver to spleen was observed between 4 h and 24 h after administration, with signal accumulation mainly in the spleen after 24 h. This was further confirmed using histology H & E (hematoxylin and eosin) staining of paraffin embedded tissue sections and Raman spectroscopy of homogenized tissue samples at different locations (Fig. 3 and S6†). Neither H & E nor Raman allowed detection of the material in the lung or kidneys, suggesting no accumulation in these tissues. Furthermore, no organ damage or other structural changes were observed in all examined organs (including lungs and kidneys) at any time point (Fig. S7†).

### Urinary excretion and urine analysis

**Fig. 4:**
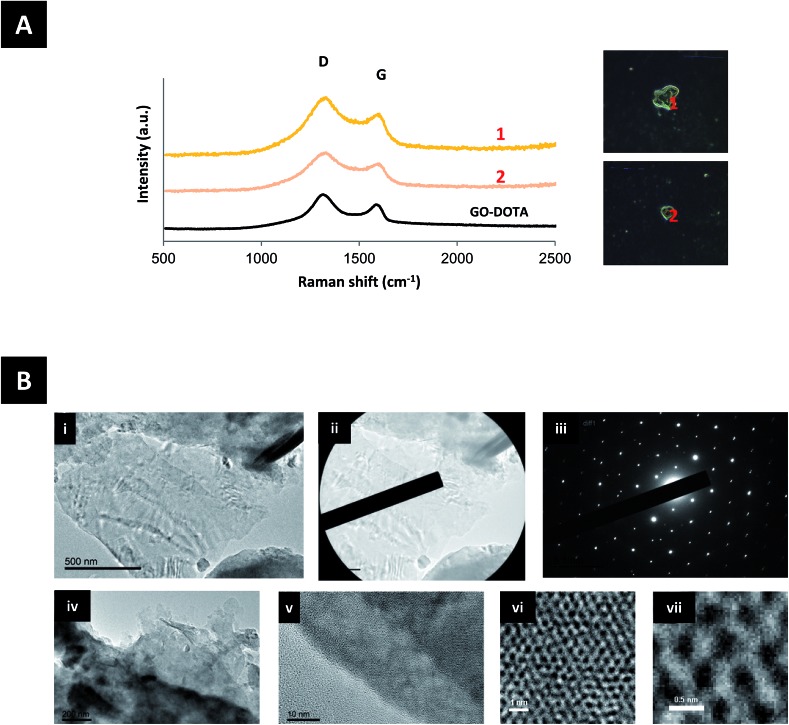
Detection of GO–DOTA in the urine of injected C57BL/6 mice 24 h post-injection as shown by: (A) Raman spectroscopy showing the GO signatures obtained from different spots within the urine sample (orange curves) and the corresponding dark-field micrographs of the samples (right). (B) HR-TEM of GO–DOTA found in the urine (i) and corresponding SAED diffraction pattern (ii and iii). Another HR-TEM image of GO–DOTA found in the urine (iv) and zoom-in images showing ordered areas (v–vii).

To further examine the extent of urinary excretions of GO–DOTA, urine samples were analyzed by Raman spectroscopy and TEM, as shown in Fig. 4 and S8.† GO–DOTA sheets were detected in the urine of injected mice by both Raman spectroscopy and HR-TEM. The Raman signature of GO–DOTA was detected in the urine of injected mice as displayed in Fig. 4A. The D band appeared wider and the *I*_D_/*I*_G_ ratio was further increased in the urine samples to 1.55 ± 0.14, however without statistical significance (Student’s *t*-test) compared to the GO–DOTA before injection. HR-TEM coupled with selected area electron diffraction (SAED) confirmed the crystalline nature of GO by the observation of the same set of six-fold symmetric diffraction spots at different locations within the samples (the GO in Fig. 4Bi contains a few layers of graphene). When increasing the magnification further, the atomically thin graphene lattice can be resolved at some locations, whereby being defective in others indicating the impact of functionalization on the hexagonal lattice (Fig. 4Bvi and vii).

## Discussion

The structural characterization of the functionalized GO derivatives by AFM and TEM (Fig. 1 & S1†) revealed that the GO sheets were increased in thickness following chemical functionalization with the DOTA chelator. This pattern has been previously reported by others after functionalization of GO with different moieties like polyethylene glycol (PEG),^21^,^37^ dextran^38^ and bovine serum albumin.^39^ Surface functionalization of GO has also been reported to reduce the lateral dimension of the sheets,^39^ also in agreement with our data (Fig. S1ii†). The presence of physically adsorbed water intercalated between GO layers makes the interpretation of the FT-IR analysis difficult and may lead to controversial assignment due to overlapping bands (Fig. 1Bi). The spectrum of GO features 4 main bands: (i) a broad band centred at 3400 cm^–1^ corresponding to O–H stretching vibrations of adsorbed water and hydroxyl functions of GO; (ii) a band at 1729 cm^–1^ attributed to the C=O stretching of carbonyl and carboxyl groups; (iii) a band at 1624 cm^–1^ related mainly to H–O–H bending vibrations of water molecules; and (iv) a band at 1374 cm^–1^ ascribed to the bending vibration of the O–H groups of GO.^40^ After functionalization of GO with TEG diamine and derivatization with DOTA, significant differences between the samples was evidenced. The band that could be assigned to the C–O–C vibration band of epoxides (at ∼1225 cm^–1^) is very small and in a region with many unassigned bands. Therefore, it is difficult to monitor the derivatization of the epoxide rings. However, the appearance of bands at 2850–2970 cm^–1^ in the GO–NH_2_ and GO–DOTA spectra, corresponding to the stretching bands of alkyl groups, demonstrate the successful attachment of the molecules.

XPS was used to determine the surface elemental composition of the GO derivatives (Fig. S2†). The nitrogen (1 s) peak of GO–NH_2_ and GO–DOTA is displayed in Fig. 1Bii. The analyses of GO–NH_2_ and GO–DOTA confirmed that nitrogen incorporation (peak at ∼400 eV) had successfully occurred after derivatization of GO with TEG diamine and DOTA–NCS. In order to offer further evidence of the functionalization of GO, we analyzed the different GO derivatives by TGA under an inert atmosphere (Fig. 1Biii). GO is thermally unstable and starts to lose mass even at temperatures lower than 100 °C. Below 150 °C, the weight loss is attributed to the volatility of water trapped between the GO layers. The higher weight loss at 230 °C is due to the elimination of labile oxygen-containing groups.^41^ The thermal profile of GO after derivatization with TEG diamine is different. The two weight loss slopes at 190 and 350 °C can be ascribed to both the elimination of oxygenated moieties and the organic functional groups arising from the newly formed carbon-bound TEG. As the thermal decomposition of TEG diamine takes place at a temperature below 200 °C, the higher degradation temperature of GO–NH_2_ is a proof of covalent bonding onto GO. Compared with the curve of GO–NH_2_, the weight loss of GO–DOTA at the end of the TG analysis (900 °C) is significantly higher. Furthermore, the thermal profile of GO–DOTA is different with a lower weight loss below 150 °C, revealing a lower amount of stacked water. This change was accompanied by a new thermal decomposition at 565 °C, which is proposed to arise from the grafted DOTA moiety, whereas the thermal decomposition of DOTA–NCS occurs at a lower temperature. As shown in the Raman spectra in Fig. 1Biv, the *I*_D_/*I*_G_ ratio of GO increased dramatically compared to graphite due to the oxidative process. After derivatization of GO with the TEG diamine and DOTA–NCS, the *I*_D_/*I*_G_ ratio did not change significantly as the reaction conditions did not lead to the introduction of further defects on the GO surface. Taken together, these data illustrate that GO was successfully covalently derivatized.

Radiolabeling of GO^17^ and chemically functionalized GO with radioactive iodine (^125^I) has been previously reported.^16^,^21^,^38^ Iodine radiolabeling requires no chelating agent attachment to the graphene material, however the method involves the use of strong oxidizing agents like chloramine T or iodogen. Moreover, such iodinated constructs are notoriously unstable, while the inherent affinity of iodine for the thyroid gland is a misleading limitation to the biodistribution profiles obtained.^38^ Radiolabeling with ^111^In has been performed previously using physical adsorption of the DTPA (diethylenetriaminepentaacetic acid) chelating agent on the surface of GO by π-stacking. Such a strategy, however, is prone to disproportion; the thickness of the GO sheets used was exceedingly large,^42^ which suggests it is within the ultrafine graphite category rather than the graphene category.^14^

Investigation of the radiolabeling efficiency and stability of GO–DOTA[^111^In] confirmed the successful covalent conjugation of DOTA on the surface of GO, and ensured that the labeling was suitable and stable in biological media for the subsequent biodistribution studies. The radiolabeling efficiency of GO–DOTA[^111^In] was found to be more than double the efficiency of the physically adsorbed control (GO + DOTA[^111^In]) (Fig. S3A†). GO–DOTA[^111^In] was stable over time up to 24 h at 37 °C, while the labeled fraction of the mixed control showed no stability over time with more than 50% release after 24 h in serum (Fig. S3A, B and E†). All non-chelated ^111^In was removed by centrifugation, until no free ^111^In signal was obtained in the samples before injection (Fig. S3B and C†). The high stability of the radiolabeled material in serum, together with the sharply different tissue distribution profiles for both control samples compared to GO–DOTA[^111^In], were considered indicative of efficient GO labeling that allowed accurate determination of GO–DOTA[^111^In] biodistribution, with negligible release of the ^111^In label from the conjugate. The aggregation of GO–DOTA sheets detected in PBS was thought to be due to the presence of a high salt concentration, while the presence of sugar (in 5% dextrose) and protein (in the serum-containing media) molecules afforded greater colloidal stability to the sample (Fig. S4†).

The biodistribution profile of the GO–DOTA[^111^In] conjugate using SPECT/CT imaging and γ-scintigraphy showed, at the early times after intravenous injection, strong signals detected in the bladder and urine of the GO–DOTA[^111^In] treated mice (Fig. 2). This profile was different from all animals injected with the controls, where complete clearance within the first hour was observed with minimal bladder residence (Fig. S5†). The urinary excretion of GO material was further confirmed by HR-TEM of the collected urine samples (Fig. 4B and S8†) showing hexagonal lattice fringes. The typical GO diffraction patterns from the SAED analysis performed at different locations within the sample showed the expected characteristic six-fold symmetry of the honeycomb organized lattice of graphene sheets at some locations with defective hexagons at others. The zoomed in images (Fig. 4vi and vii) showed some defect-free areas interspersed with defected areas. This has been reported for chemically derived graphene materials that originate from the oxidation-reduction treatment.^43^

The Raman signature of GO–DOTA detected in the urine of injected mice further confirmed the intact sheet urinary excretion (Fig. 4A). The increase in the width of the D band and ratio of the *I*_D_/*I*_G_ bands may be indicative of further defects introduced on the surface of the GO–DOTA[^111^In] during transport through blood circulation and excretion, however the absence of statistical significance compared to the GO–DOTA before injection warrants the need for further work. Increase in *I*_D_/*I*_G_ band ratios and widths of D bands of intravenously injected GO has been reported previously in different organs including lung, liver, kidney and spleen starting at 24 h post-injection, but was attributed to biodegradation processes within different tissue-bound macrophages.^27^

Previously, only indirect evidence of renal clearance has been reported after intravenous administration of small GO sheets functionalized with dextran^38^ or PEG.^16^,^44^ That was due to the reduced dimensions of those sheets that were thought to easily cross the renal filtration slit (<40 nm).^44^,^45^ Excretion of GO–DOTA sheets through the renal pathway (Fig. 4 and S8†) has not been reported before, even though no such GO derivative has been previously synthesized and studied. A possible explanation could be the folding or rolling of the thin sheets into smaller dimensions^46^–^48^ during blood circulation. In this way, a significant fraction of the GO sheets could translocate the glomerular filtration system in a manner similar to that described previously for chemically functionalised single and multi-walled carbon nanotubes.^49^,^50^ Another mechanism could be the sliding of the thin GO sheets perpendicularly through the cellular membranes as suggested *in vitro*.^51^ Further investigations are certainly warranted to determine the excretion mechanism of such chemically functionalized GO sheets.

Other carbon nanostructures, such as chemically functionalized carbon nanotubes, have been reported to be able to excrete intact in the urine of injected mice.^52^,^53^ Given their average length of 200–500 nm, they were thought to rapidly eliminate through the glomerular filter by alignment with blood flow and perpendicularly translocating through the glomerular filter.^49^,^54^ Hydroxyl-functionalized fullerenes were also reported to be rapidly excreted through the urinary tract of rats and rabbits, while carboxylic acid-functionalized fullerenes were retained mainly in the liver 48 h post-injection.^52^,^55^ It is becoming evident that different carbon nanostructures, with varying dimensions, shape, surface, degree of functionalization and individualization follow different *in vivo* pathways, that determine their tissue affinity, accumulation and excretion.^31^,^49^ We postulate that for graphene materials three fundamental parameters are highly important in determining the biological fate. Lateral dimension and thickness (*i.e.* layer number) may alter the stiffness and flexibility of the material, which will have subsequently a great impact on interactions with tissues and cells. Lastly, the degree and nature of the functionalization (the hydrophilic/hydrophobic surface character) of the material^14^,^30^ may have an impact on the interactions with proteins of the extracellular matrix or blood plasma.

In this study, GO was observed to translocate from liver to spleen, evidenced by the reduction of signal in the liver with concomitant increased or persistent signal in the spleen (Fig. 2 and 3). Tissue distribution of intravenously injected graphene derivatives has been reported previously using material with different surface functionalities. PEG-functionalized GO was reported to accumulate in the RES organs (liver, spleen) with slow clearance over time in urine and faeces, and renal clearance that was attributed to the smaller sized (<40 nm) sheets able to cross the glomerular filtration slit. Accumulation in the RES was attributed to larger sheets uptaken by macrophages, however, no such experimental evidence was shown.^16^ Another study, using both PEG-functionalized GO and rGO constructs, demonstrated maximum organ accumulation in the liver for all constructs, regardless of their significant differences in size and surface characteristics.^56^ In a different study, high kidney accumulation was reported after 24 h from i.v. injected PEG-functionalized small GO (lateral dimension 10–50 nm), with lower accumulation in the lung, liver and spleen.^44^ In a study using dextran-functionalized GO, mainly accumulation in the liver was reported, with both faecal and urinary excretions,^38^ while NOTA (1,4,7-triazacyclononane-triacetic acid)-functionalized GO-PEG was also shown to accumulate in the liver.^57^,^58^ On the other hand, non-functionalized GO of large lateral dimensions (>500 nm) administered intravenously in mice has been reported to accumulate to a large extent in the lung.^17^,^59^,^60^ However, we speculate that this was mainly due to aggregation among flakes in these GO dispersions. Smaller GO sheets (100–500 nm) were shown to accumulate mainly in the liver.^17^

All the above studies suggested that the dimensions and surface groups are critical to determine the fate of GO after intravenous administration. The two-phase biodistribution profile of the GO sheets studied here can be related to the wide thickness distribution of the GO sheets. We speculate that thin, flexible sheets may tend to roll, fold or slid and cross the glomerular filtration barrier, while thicker sheets may favour entrapment and uptake by spleen cells. The GO–DOTA[^111^In] construct mainly accumulated in the splenic red pulp (Fig. 3B and S6†). This may involve immune cells (*e.g.* engulfment by spleen-bound macrophages or monocytes) since the splenic red pulp is rich with such cells. The low Raman signal from the faeces samples suggested no involvement of biliary excretion or metabolism by hepatocytes in the time-frame of our experiments.

Our work suggests that further investigations are required to determine the cell types involved in these interactions and whether cellular uptake of the GO sheets occurred by tissue-residing macrophages, or initially by circulating monocytes that subsequently migrate into the spleen. It has been previously reported that medium-sized nanoparticles (10–300 nm in diameter) tend to accumulate in the liver and spleen, as these organs contain high numbers of macrophages.^61^ Such findings have been proposed as a strategy to label and image macrophages in cancer, atherosclerosis, myocardial infarction and stroke, since macrophage infiltration is involved in all of these pathologies. Splenic drug delivery could be another potential application of these GO constructs with the aim to effectively transport drugs or enzymes to the spleen for potential immunostimulatory interventions.^62^

In conclusion, thin GO sheets were successfully conjugated with the DOTA chelator to offer high chemical and radiolabeling stability. Intravenous administration of the moderately thicker GO–DOTA flakes led to rapid and significant urinary excretion followed by gradual accumulation in the spleen. The detection of intact hexagonal lattices in the excreted urine indicated GO clearance that has not been previously reported. These findings provide further understanding of the kinetics and barrier interactions of the thin functionalized GO sheets after intravenous administration in mice. Such findings have important implications in the future design of graphene-based materials for imaging and therapeutic purposes, as well as the determination of their safety profile.

## Experimental

### Materials

Flake graphite was purchased from Barnwell. All solvents and analytical grade H_2_SO_4_ were purchased from Fisher Scientific (UK). All other chemicals including KMnO_4_, NaNO_3_, H_2_O_2_, 2,2′-(ethylenedioxy)bis(ethylamine), and DOTA were purchased from Sigma-Aldrich. 2-(4-isothiocyanatobenzyl)-1,4,7,10-tetraazacyclododecane-1,4,7,10-tetraacetic acid (DOTA–NCS) was purchased from Macrocyclics. The filtration and dialysis membranes were purchased from Millipore and Spectrum Laboratories, Inc., respectively.

### Chemical synthesis of graphene oxide

GO was prepared by the modified Hummers' method described in Ali-Boucetta *et al.*^20^ Briefly, 0.4 g of graphite was mixed with 0.2 g of NaNO_3_ and 9.2 mL of 96% H_2_SO_4_. KMnO_4_ (1.2 g) was then added slowly after obtaining a homogenous mixture. The temperature was monitored carefully during the reaction and was kept between 98–100 °C. The mixture was further diluted with 50 mL of deionized H_2_O and 3% H_2_O_2_ was added gradually for the reduction of the residual KMnO_4_, MnO_2_ and Mn_2_O_7_ to soluble MnSO_4_ salts. The resulting suspension was purified by several centrifugation steps until the pH of the supernatant was around 7 and a viscous orange/brown layer of pure GO appeared on top of the oxidation by-products. This was the fraction of pure GO that was used for later experiments.

### Preparation of GO–NH_2_ and GO–DOTA

To a solution of GO (17 mg) in deionized water (17 mL) was added triethylene glycol diamine (2,2′-(ethylenedioxy)bis(ethylamine), 350 μL). The reaction mixture was stirred for 3 days. The solution was then filtered on an Omnipore® polytetrafluoroethylene (PTFE) membrane (0.1 μm). The solid was dispersed in DMF, sonicated for 1 min and filtered again. This procedure was repeated with DMF, methanol (twice), and dichloromethane to give GO–NH_2_. The solid was dispersed in deionized water and dialyzed against deionized water using a dialysis membrane of MWCO 12–14 000 Da.

To a suspension of GO–NH_2_ (14 mg) in deionized water (7 mL) were added sodium bicarbonate (1.2 mg) and DOTA–NCS (5.7 mg). The reaction mixture was stirred for 3 days. The dispersion was then filtered on a PTFE 0.1 μm membrane. The solid was dispersed in DMF, sonicated for 1 min and filtered again. This procedure was repeated with methanol (twice) and dichloromethane. The dispersion was dialyzed against deionized water using a 12–14 000 Da MWCO dialysis membrane.

### Preparation of control sample GO + DOTA

To a solution of GO (130 μg) in water (130 μL) was added DOTA (130 μg). The reaction mixture was sonicated for 1 min and stirred for 1 day. The suspension was then dialyzed against deionized water using a 12–14 000 Da MWCO dialysis membrane.

### Preparation of ^111^In labeled GO–DOTA

GO–DOTA was diluted with an equal volume of 0.2 M ammonium acetate buffer pH 5.5, to which 2–20 MBq as ^111^InCl_3_ was added. The indium was left to react with the GO–DOTA for 60 min at 60 °C, after which the reaction was quenched by the addition of 0.1 M EDTA chelating solution.

### Radiolabeling efficiency of GO–DOTA[^111^In]

To determine the labeling efficiency, aliquots of each final product were diluted five folds in PBS and then 1 μL was spotted on silica gel impregnated glass fibre sheets (PALL Life Sciences, UK). The strips were developed with a mobile phase of 50 mM EDTA in 0.1 M ammonium acetate and allowed to dry before analysis. This was then developed and the autoradioactivity quantitatively counted using a Cyclone phosphor detector (Packard Biosciences, UK). The immobile spot on the TLC strips indicated the percentage of radiolabeled GO–DOTA, while free EDTA[^111^In] or DOTA[^111^In] were seen as the mobile spots near the solvent front. DOTA conjugation efficiency studies were performed using a modified running buffer containing no EDTA at pH 9, prepared from methanol and ammonia 3.5% solution at a ratio of 1 : 1 to precipitate any free ^111^In.

### Radiolabeling and colloidal stability of GO–DOTA

To determine the stability of the labeled GO–DOTA[^111^In], aliquots of each final product were diluted five folds either in PBS or mouse serum and then incubated at 37 °C over 24 h. At different time-points (0, 1 and 24 h), 1 μL of the aliquots was spotted on silica gel impregnated glass fibre sheets and then developed, and quantified as described above. To determine the colloidal stability of the GO–DOTA flakes, five-fold dilution of the sample was carried out in dextrose 5%, PBS, or serum (50%) and kept at 37 °C up to 4 h. Dynamic light scattering (DLS) was performed at (0 h, 1 h and 4 h) using the Malvern Zetasizer Nano ZS (UK). Measurements were performed after dilution with water in 1 mL final total volume using disposable cuvettes (Sartorius Stedim, Epsom, UK). Default instrument settings and automatic analysis were used for all measurements. Independent triplicate measurements were carried out. It should be noted that DLS is thought to offer an approximate, semi-quantitative determination of the mean particle diameter in the dispersion, but is not the ideal technique for non-spherical particles.

### Physicochemical and structural characterization methods

All samples were characterized by TEM using a BioTwin electron microscope (Philips/FEI), Tecnai 12 instrument operated at 120 kV accelerating voltage. One drop of sample was placed on a formvar/carbon coated copper grid. Filter paper was used to remove the excess of material. AFM was carried out using a multimode AFM on the tapping-mode with an J-type scanner, Nanoscope V controller, Nanoscope v8.15 control software (Veeco, Cambridge, UK) and an Olympus high aspect ratio etched silicon probe (OTESPA) with a nominal spring constant of 42 N m^–1^ (Bruker AXS S.A.S., France). Cantilever oscillation varied between 300 and 350 kHz whilst the drive amplitude was determined by the Nanoscope (v8.15) software. Height images were captured at a scan rate of 1.5 Hz. Data was first-order flattened using the Nanoscope software prior to image export. Images were taken in air, by depositing 20 μL of the graphene dispersion on a freshly cleaved mica surface (Agar Scientific, Essex, UK) coated with poly-lysine 0.01% (Sigma) and allowed to adsorb for 30 s. Excess unbound material was removed by washing with filtered distilled water, and then allowed to dry in air. Size distributions were carried out using ImageJ software, to measure the lateral dimension of individual graphene sheets. Sheet thickness distribution was determined from AFM height sections. Both size and thickness distributions were based on counting more than 100 sheets from several AFM images. The Kaiser test was performed according to a procedure described in ref. 63. Raman spectra of samples were recorded after preparing the aqueous dispersions and drop casting them on glass slides and then evaporation of the water. Measurements were carried out using a 50× objective at 780 nm laser excitation using a Renishaw micro-Raman spectrometer. Raman spectra were measured at several different locations and three different spectra were collected for each location. FT-IR spectra were measured on a Perkin Elmer Spectrum One ATR-FT-IR spectrometer. TGA was performed using a TGA1 (Mettler Toledo) apparatus from 30 °C to 900 °C with a ramp of 10 °C min^–1^ under N_2_ using a flow rate of 50 mL min^–1^ and platinum pans. XPS analyses were performed with a MULTILAB 2000 (THERMO) spectrometer equipped with an anode using Al Kα radiation (*hν* = 1486.6 eV) during 10 min of acquisition in order to achieve a good signal-to-noise ratio. The C (1s) photoelectron binding energy was set at 284.6 ± 0.2 eV relatively to the Fermi level and used as reference for calibrating the other peak positions.

### Animal handling procedures

Six- to eight-week-old C57BL/6 mice were obtained from Harlan (Oxfordshire, UK), allowed to acclimatize for 1 week and were given food and water for the duration of the experiments. All experiments were conducted with prior approval from the UK Home Office.

### Single photon emission computed tomography (SPECT/CT)

Mice were anaesthetized by isofluorane inhalation. Each animal was injected *via* the tail vein with 200 μL containing 50 μg of GO–DOTA[^111^In] labeled with approximately 5–6 MBq. At different time points after injection (*t* = 1, 4 and 24 h), mice were imaged using the Nano-SPECT/CT scanner (Bioscan, USA). SPECT images were obtained in 24 projections over 40–60 min using a four-head scanner with 1.4 mm pinhole collimators. CT scans were taken at the end of each SPECT acquisition and all images were reconstructed with MEDISO software (Medical Imaging Systems). Fusion of SPECT and CT images was carried out using the PMOD software.

### Gamma scintigraphy

For more quantitative assessment of tissue biodistribution, a cut and count study was carried out. Mice were anaesthetized by isofluorane inhalation. Each animal was injected *via* the tail vein with 200 μL containing 50 μg of GO–DOTA[^111^In] containing approximately 1–2 MBq. Mice were sacrificed at 1, 4 and 24 h after injection, and blood and all major organs and tissues were collected including, heart, lungs, liver, spleen, kidneys, muscle, skin and bone. Urine and faeces were pooled and collected after 24 h. Each sample was weighted and counted on a gamma Counter (Perkin Elmer, USA), together with a dilution of the injected dose with dead time limit below 60%. The percentage injected dose per gram tissue was calculated, using four different mice for each time point.

### Histological analysis

Lungs, liver, spleen and kidneys were extracted from mice at different time points (1, 4 and 24 h) and fixed with 4% paraformaldehyde. This was followed by paraffin embedding of sections at known orientations. Sections of 5 μm were stained with hematoxylin and eosin (H & E) and imaged using a LEICA DM 2000 optical microscope equipped with LEICA application suit v3.2.0 software coupled to LEICA DF295 camera.

### Purification of urine samples

The urine samples were dialyzed against deionized water using a 300 000 MWCO dialysis membrane and then lyophilized.

### Raman microscopy of tissue and urine samples

Tissues were physically homogenized and placed on glass slides. Urine samples were purified as described above and drop-casted and dried on the glass slide. Spectra were measured at several different locations within the tissue and urine samples. An average of three different readings was collected for each location.

### High resolution transmission electron microscopy (HR-TEM) of urine samples

HR-TEM and SAED analyses have been performed on a JEOL 2100F TEM/STEM electron microscope operating at 200 kV.

### Statistics

Values are mean ± SD (*n* = 3–4). Statistical significance was evaluated by Student's *t*-test (*p* < 0.05 *).

## Supplementary Material

Supplementary information

## References

[cit1] Geim A. K. (2009). Science.

[cit2] Novoselov K. S., Fal′ko V. I., Colombo L., Gellert P. R., Schwab M. G., Kim K. (2012). Nature.

[cit3] Pan Y., Sahoo N. G., Li L. (2012). Expert Opin. Drug Delivery.

[cit4] Krishna K. V., Ménard-Moyon C., Verma S., Bianco A. (2013). Nanomedicine.

[cit5] Bitounis D., Ali-Boucetta H., Hong B. H., Min D.-H., Kostarelos K. (2013). Adv. Mater..

[cit6] Shen H., Zhang L., Liu M., Zhang Z. (2012). Theranostics.

[cit7] Loh K. P., Bao Q., Eda G., Chhowalla M. (2010). Nat. Chem..

[cit8] Feng L., Liu Z. (2011). Nanomedicine.

[cit9] Ferrari A. C., Meyer J. C., Scardaci V., Casiraghi C., Lazzeri M., Mauri F., Piscanec S., Jiang D., Novoselov K. S., Roth, S., Geim A. K. (2006). Phys. Rev. Lett..

[cit10] Bendali A., Hess L. H., Seifert M., Forster V., Stephan A.-F., Garrido J. A., Picaud S. (2013). Adv. Healthcare Mater..

[cit11] Liu Y., Dong X., Chen P. (2012). Chem. Soc. Rev..

[cit12] Seidlits S. K., Lee J. Y., Schmidt C. E. (2008). Nanomedicine.

[cit13] Bianco A., Cheng H.-M., Enoki T., Gogotsi Y., Hurt R. H., Koratkar N., Kyotani T., Monthioux M., Park C. R., Tascon J. M.D., Zhang J. (2013). Carbon.

[cit14] Wick P., Louw-Gaume A. E., Kucki M., Krug H. F., Kostarelos K., Fadeel B., Dawson K. A., Salvati A., Vázquez E., Ballerini L., Tretiach M., Benfenati F., Flahaut E., Gauthier L., Prato M., Bianco A. (2014). Angew. Chem. Int. Ed..

[cit15] Wilson N. R., Pandey P. A., Beanland R., Young R. J., Kinloch I. A., Gong L., Liu Z., Suenaga K., Rourke J. P., York S. J., Sloan J. (2009). ACS Nano.

[cit16] Yang K., Wan J., Zhang S., Zhang Y., Lee S.-T., Liu Z. (2011). ACS Nano.

[cit17] Liu J.-H., Yang S.-T., Wang H., Chang Y., Cao A., Liu Y. (2012). Nanomedicine.

[cit18] Qi W., Li Z., Bi J., Wang J., Wang J., Sun T., Guo Y., Wu W. (2011). J. Nanopart. Res..

[cit19] Qu G., Wang X., Liu Q., Liu R., Yin N., Ma J., Chen L., He J., Liu S., Jiang G. (2013). J. Environ. Sci..

[cit20] Ali-Boucetta H., Raveendran-Nair R., Servant A., Van den Bossche J., Kostarelos K. (2012). Adv. Healthcare Mater..

[cit21] Yang K., Gong H., Shi X., Wan J., Zhang Y., Liu Z. (2013). Biomaterials.

[cit22] Yan L., Wang Y., Xu X., Zeng C., Hou J., Lin M., Xu J., Sun F., Huang X., Dai L., Lu F., Liu Y. (2012). Chem. Res. Toxicol..

[cit23] Zhang X., Yin J., Peng C., Hu W., Zhu Z., Li W., Fan C., Huang Q. (2011). Carbon.

[cit24] Duch M. C., Budinger G. R. S., Liang Y. T., Soberanes S., Urich D., Chiarella S. E., Campochiaro L. A., Gonzalez A., Chandel N. S., Hersam, M. C., Mutlu G. M. (2011). Nano Lett..

[cit25] Schinwald A., Murphy F. A., Jones A., MacNee W., Donaldson K. (2011). ACS Nano.

[cit26] Ma-Hock L., Strauss V., Treumann S., Küttler K., Wohlleben W., Hofmann T., Gröters S., Wiench K., van Ravenzwaay B., Landsiedel R. (2013). Part. Fibre Toxicol..

[cit27] Girish C. M., Sasidharan A., Gowd G. S., Nair S., Koyakutty M. (2013). Adv. Healthcare Mater..

[cit28] Bianco A. (2013). Angew. Chem., Int. Ed..

[cit29] Kostarelos K., Novoselov K. S. (2014). Science.

[cit30] Bussy C., Jasim D. A., Lozano N., Terry D., Kostarelos K. (2015). Nanoscale.

[cit31] Al-Jamal K. T., Nunes A., Methven L., Ali-Boucetta H., Li S., Toma F. M., Herrero M. A., Al-Jamal W. T., ten Eikelder H. M. M., Foster J., Mather S., Prato M., Bianco, A., Kostarelos K. (2012). Angew. Chem., Int. Ed..

[cit32] Bussy C., Ali-Boucetta H., Kostarelos K. (2012). Acc. Chem. Res..

[cit33] Dreyer D. R., Park S., Bielawski C. W., Ruoff R. S. (2010). Chem. Soc. Rev..

[cit34] Eigler S., Hu Y., Ishii Y., Hirsch A. (2013). Nanoscale.

[cit35] Thomas H. R., Marsden A. J., Walker M., Wilson N. R., Rourke J. P. (2014). Angew. Chem., Int. Ed. Engl..

[cit36] Kaiser E., Colescott R. L., Bossinger C. D., Cook P. I. (1970). Anal. Biochem..

[cit37] Zhang W., Wang C., Li Z., Lu Z., Li Y., Yin J.-J., Zhou Y.-T., Gao X., Fang Y., Nie G., Zhao Y. (2012). Adv. Mater..

[cit38] Zhang S., Yang K., Feng L., Liu Z. (2011). Carbon.

[cit39] Li Y., Feng L., Shi X., Wang X., Yang Y., Yang K., Liu T., Yang G., Liu Z. (2014). Small.

[cit40] Szabó T., Berkesi O., Dékány I. (2005). Carbon.

[cit41] Jung I., Field D. A., Clark N. J., Zhu Y., Yong D., Piner R. D., Stankovich S., Dikin D. A., Geisler H., Ventrice, Jr. C. A., Ruoff R. S. (2009). J. Phys. Chem. C.

[cit42] Cornelissen B., Able S., Kersemans V., Waghorn P. A., Myhra S., Jurkshat K., Crossley A., Vallis K. A. (2013). Biomaterials.

[cit43] Gómez-Navarro C., Meyer J. C., Sundaram R. S., Chuvilin A., Kurasch S., Burghard M., Kern K., Kaiser U. (2010). Nano Lett..

[cit44] Yang K., Zhang S., Zhang G., Sun X., Lee S.-T., Liu Z. (2010). Nano Lett..

[cit45] Lacerda L., Soundararajan A., Singh R., Pastorin G., Al-Jamal K. T., Turton J., Frederik P., Herrero M. A., Li S., Bao A., Emfietzoglou D., Mather S., Phillips W. T., Prato M., Bianco A., Goins B., Kostarelos K. (2008). Adv. Mater..

[cit46] Ivanovskaya V. V., Wagner P., Zobelli A., Suarez-Martinez I., Yaya A. and Ewels C. P., Graphene Edge Structures: Folding, Scrolling, Tubing, Rippling and Twisting, GraphITA 2011, Springer, Heidelberg, Berlin, 2012, pp. 75–85.

[cit47] Meyer J. C., Geim A. K., Katsnelson M. I., Novoselov K. S., Booth T. J., Roth S. (2007). Nature.

[cit48] Patra N., Wang B., Král P. (2009). Nano Lett..

[cit49] Lacerda L., Herrero M. A., Venner K., Bianco A., Prato M., Kostarelos K. (2008). Small.

[cit50] Lacerda L., Russier J., Pastorin G., Herrero M. A., Venturelli E., Dumortier H., Al-Jamal K. T., Prato M., Kostarelos K., Bianco A. (2012). Biomaterials.

[cit51] Russier J., Treossi E., Scarsi A., Perrozzi F., Dumortier H., Ottaviano L., Meneghetti M., Palermo V., Bianco A. (2013). Nanoscale.

[cit52] Singh R., Pantarotto D., Lacerda L., Pastorin G., Klumpp C., Prato M., Bianco A., Kostarelos K. (2006). Proc. Natl. Acad. Sci. U. S. A..

[cit53] Ruggiero A., Villa C. H., Bander E., Rey D. A., Bergkvist M., Batt C. A., Manova-Todorova K., Deen W. M., Scheinberg, D. A., McDevitt M. R. (2010). Proc. Natl. Acad. Sci. U. S. A..

[cit54] Kostarelos K. (2010). Nat. Mater..

[cit55] Cagle D. W., Kennel S. J., Mirzadeh S., Alford J. M., Wilson L. J. (1999). Proc. Natl. Acad. Sci. U. S. A..

[cit56] Yang K., Wan J., Zhang S., Tian B., Zhang Y., Liu Z. (2012). Biomaterials.

[cit57] Hong H., Zhang Y., Engle J. W., Nayak T. R., Theuer C. P., Nickles R. J., Barnhart T. E., Cai W. (2012). Biomaterials.

[cit58] Hong H., Yang K., Zhang Y., Engle J. W., Feng L., Yang Y., Nayak T. R., Goel S., Bean J., Theuer C. P., Barnhart T. E., Liu Z., Cai W. (2012). ACS Nano.

[cit59] Wang K., Ruan J., Song H., Zhang J., Wo Y., Guo S., Cui D. (2011). Nanoscale Res. Lett..

[cit60] Singh S. K., Singh M. K., Nayak M. K., Kumari S., Shrivastava S., Grácio, J. J. A., Dash D. (2011). ACS Nano.

[cit61] Ralph W., Matthias N., Mikael J. P. (2014). Nat. Mater..

[cit62] Patil R. R., Gaikwad R. V., Samad A., Devarajan P. V. (2008). J. Biomed. Nanotechnol..

[cit63] Ménard-Moyon C., Fabbro C., Prato M., Bianco A. (2011). Chem. - Eur. J..

