# Supplementary material for: Tissue distribution and urinary excretion of intravenously administered chemically functionalized graphene oxide sheets
Source: Chem Sci. 2015 Apr 14;6(7):3952–64. doi: 10.1039/c5sc00114e (PMC5497267; doi:10.1039/c5sc00114e)
Supplement: Supplementary file 1 [file SC-006-C5SC00114E-s001.pdf]

Electronic Supplementary Material (ESI) for Chemical Science.
This journal is © The Royal Society of Chemistry 2015

Supporting Information

Tissue Distribution and Urinary Excretion of Intravenously Administered Chemically Functionalized Graphene Oxide Sheets

Dhifaf A. Jasim, Cécilia Ménard-Moyon, Dominique Bégin, Alberto Bianco, Kostas Kostarelos**

Supporting Figures

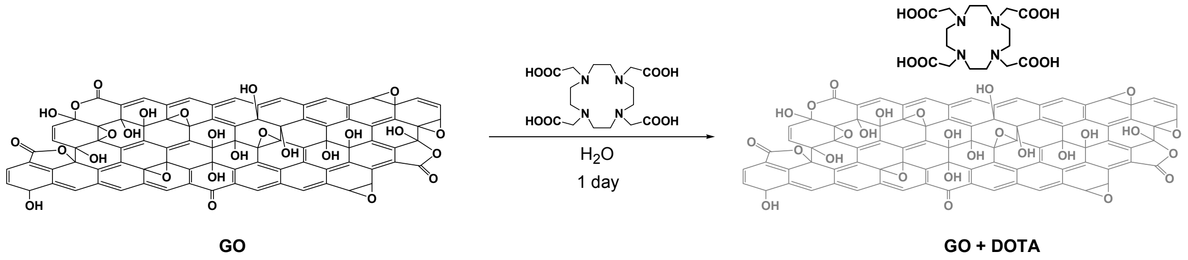

Scheme S1: Preparation of control sample GO + DOTA. GO was simply mixed with DOTA and treated to remove excess DOTA which was not physically adsorbed onto GO.

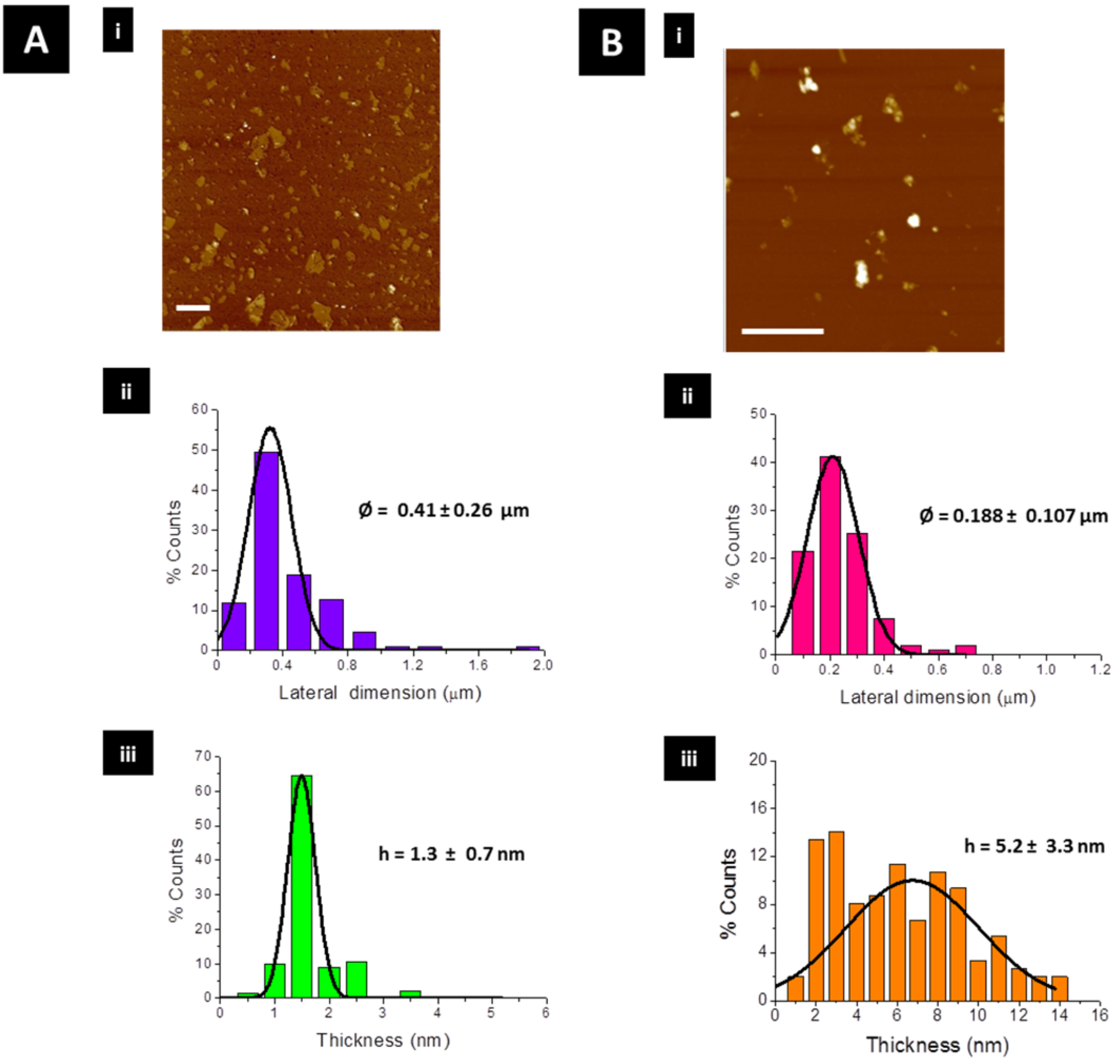

Supporting Figure 1: AFM of (A) GO and (B) GO-DOTA. (i) AFM images; (ii) lateral size distribution measured by counting the lateral dimension; (iii) thickness distribution obtained from height sections. Percent counts were obtained by counting more than 100 sheets obtained from several images per sample. Scale bars are 1μm.

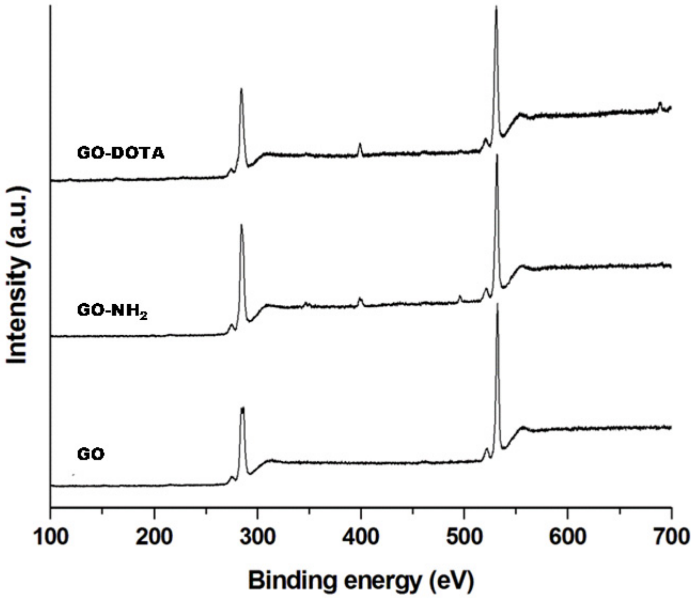

Supporting Figure 2: XPS of GO, GO-NH_2 and GO-DOTA showing the appearance of the N (1s) peak at ~400 eV, compared to XPS of GO.

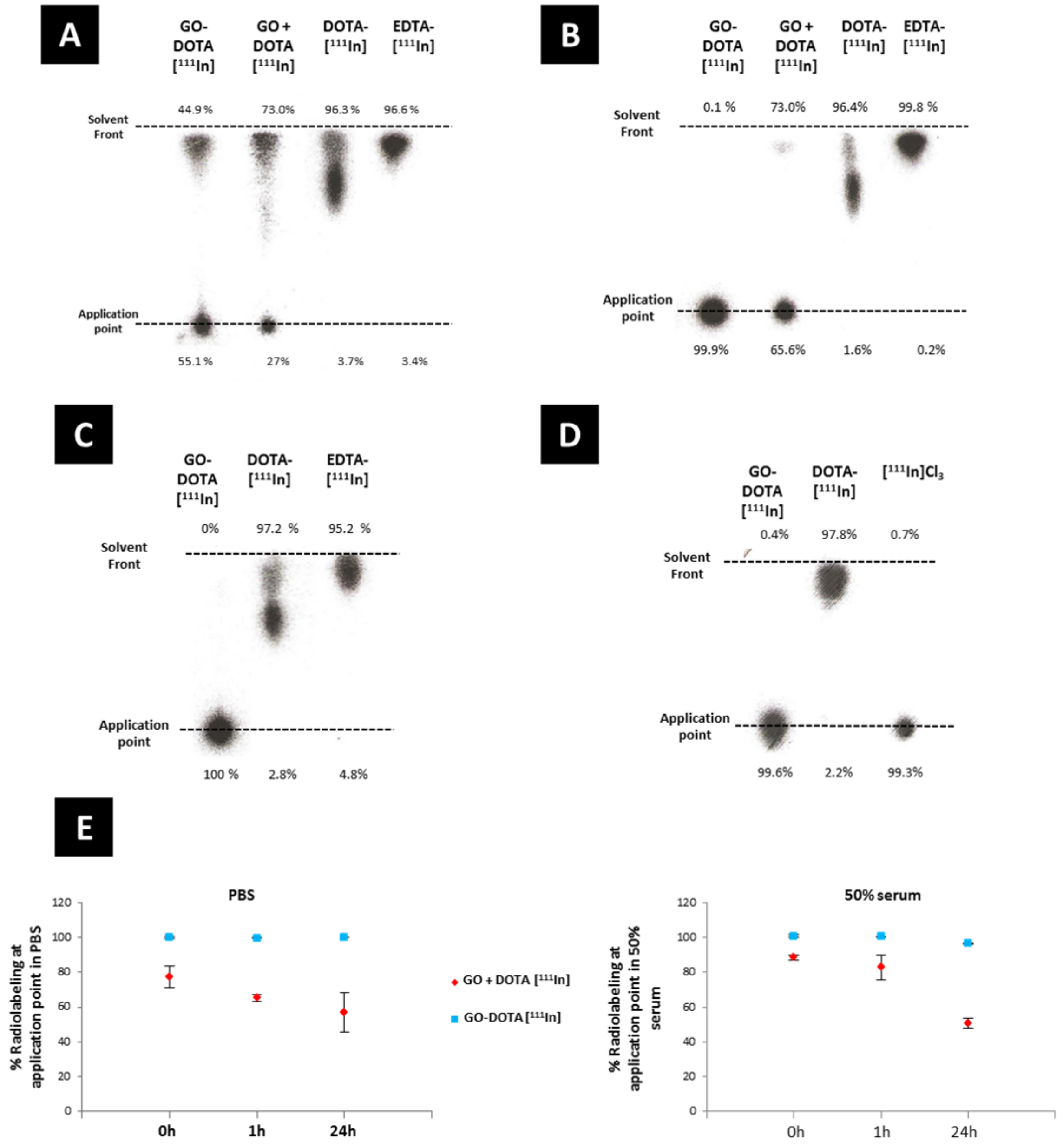

Supporting Figure 3: Thin layer chromatography of radiolabeled constructs showing: (A) labeling efficiency after radiolabeling with ^{111}In of the chemically conjugated (GO-DOTA) compared to the physically adsorbed control (GO + DOTA); (B) removal of free ^{111}In from GO-DOTA[^{111}In] sample by centrifugation for 30 min compared to the physically adsorbed control (GO + DOTA[^{111}In]); (C) no free (unbound) ^{111}In was detected before injecting samples in mice; (D) DOTA conjugation efficiency detected by using a modified running buffer at pH 9; (E) radiolabeling stability up to 24h of the chemically conjugated sample (GO-DOTA[^{111}In]) compared to the physically adsorbed control (GO + DOTA[^{111}In]) at 37°C, in both 50% serum and PBS.

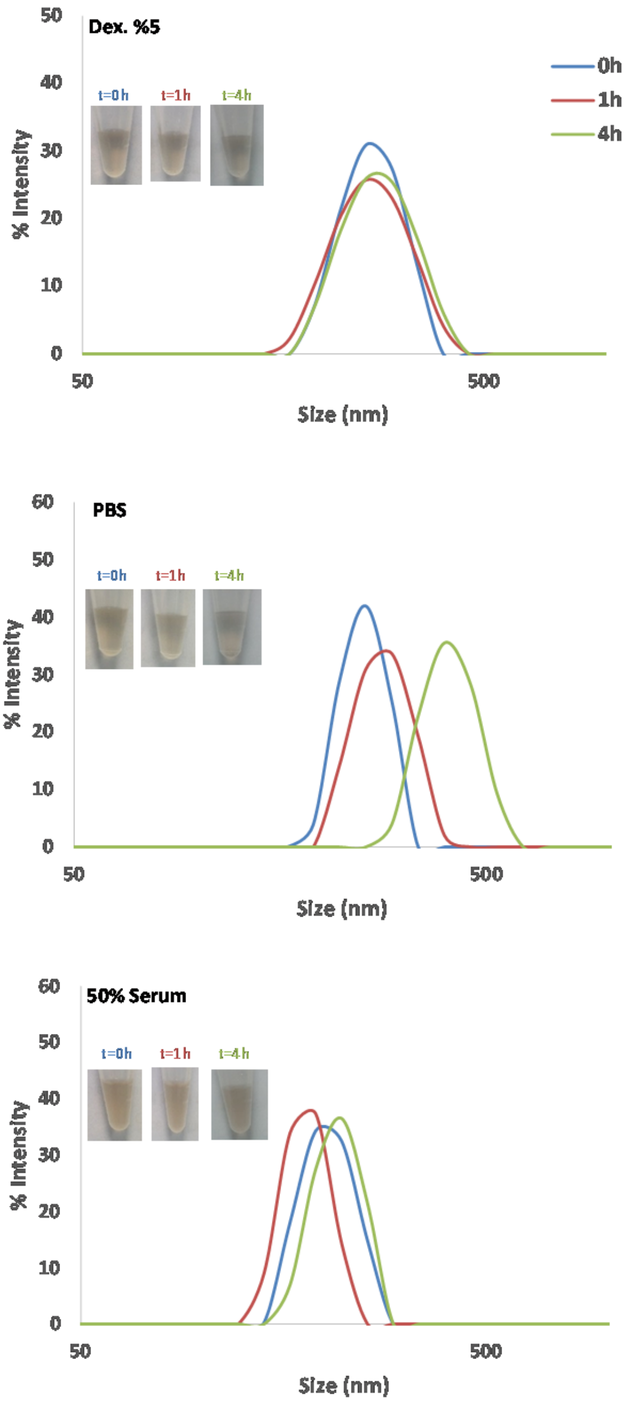

Supporting Figure 4: Colloidal stability of the GO-DOTA sheets dispersed in Dextrose, PBS and serum (50%) (from top to bottom) for t=0, 1h and 4h. The mean size of the dispersed material was measured using dynamic light scattering using Malvern Zetasizer Nano ZS (UK).

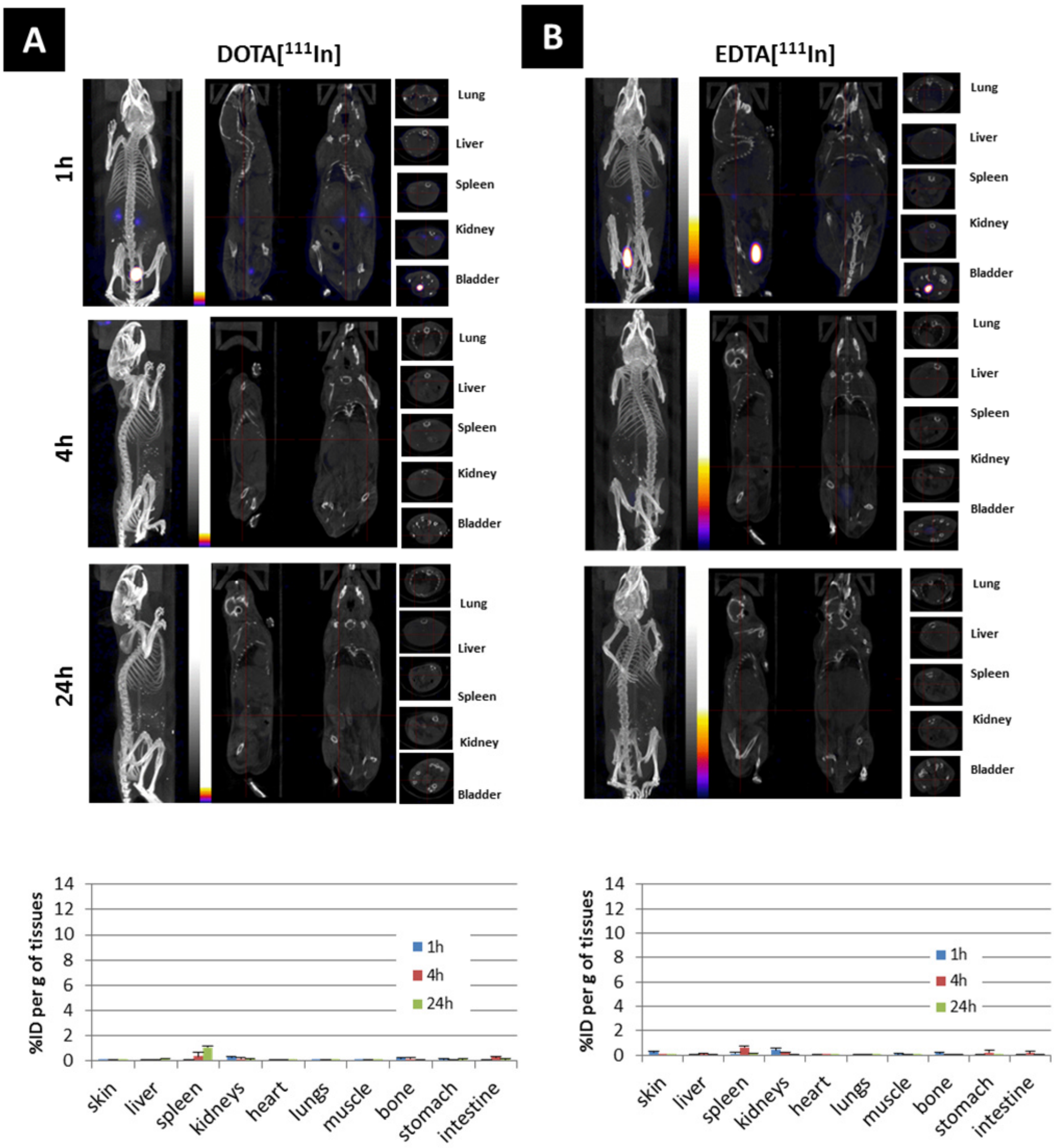

Supporting Figure 5: Biodistribution of **(A)** DOTA[^{111}In] and **(B)** EDTA[^{111}In]. Whole body SPECT/CT imaging of a C57BL/6 mouse injected with DOTA[^{111}In] and EDTA[^{111}In], respectively, imaged at different time points (1, 4, 24 h) showing from left to right whole body, sagittal, coronal and transverse views. Gamma scintigraphy bar charts showing organ biodistribution (n=4/group).

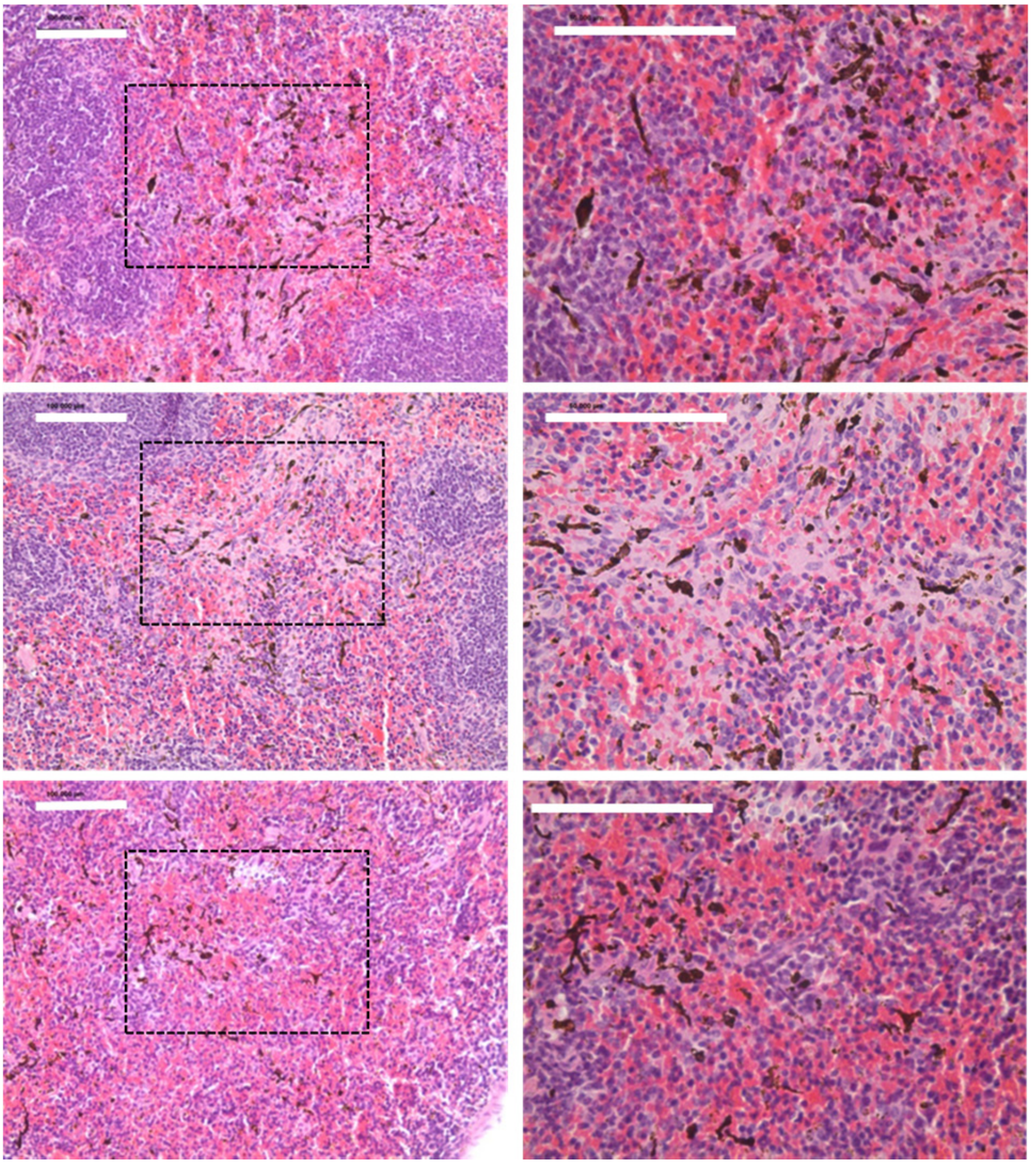

Supporting Figure 6: Histology using H & E staining of spleen sections showing GO-DOTA accumulation at 24h post-injection. Right hand-side images are higher magnification images of the boxes on the left hand-side sections. All scale bars are 100 μm.

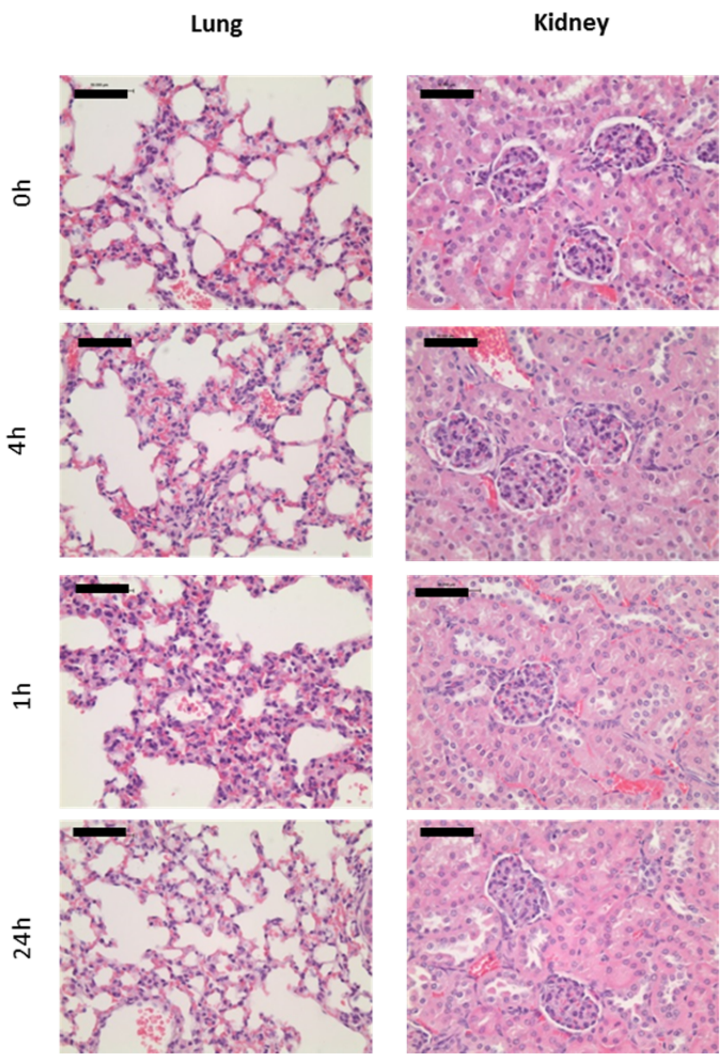

Supporting Figure 7: Histology using H & E staining of lung (left) and kidney (right) tissue sections from mice injected with 50 µg of GO-DOTA chelated with non-radioactive $InCl_3$ after 1, 4 and 24h compared to uninjected mice at time 0h. All scale bars are 50 µm.

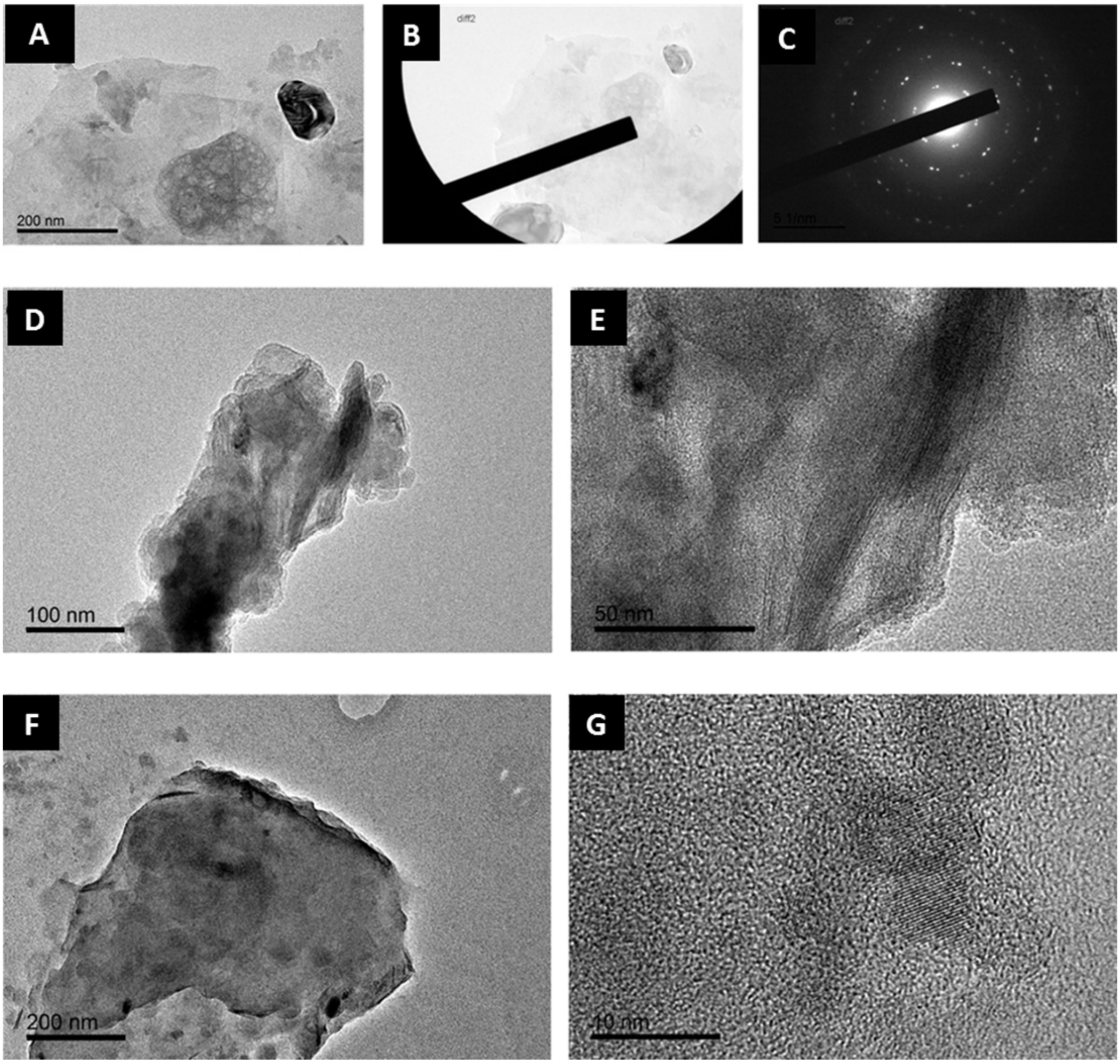

Supporting Figure 8: HR-TEM of GO-DOTA found in the urine after 24h **(A)** and their corresponding SAED diffraction patterns **(B, C)**. Other HR-TEM images of GO-DOTA found in the urine **(D, E)** and corresponding magnification images showing ordered areas **(F, G)**.
